# Effect of Trace Sc Addition on Microstructure and Mechanical Properties of Al-Zn-Mg-Cu-Zr Alloy

**DOI:** 10.3390/ma18030648

**Published:** 2025-01-31

**Authors:** Yuchen Huang, Linfei Xia, Huabing Yang, Chengguo Wang, Yuying Wu, Xiangfa Liu

**Affiliations:** 1 Key Laboratory for Liquid-Solid Structure Evolution and Processing of Materials, Ministry of Education, Shandong University, Jinan 250061, China; 202334198@mail.sdu.edu.cn (Y.H.); lfxia@mail.sdu.edu.cn (L.X.); xfliu@sdu.edu.cn (X.L.); 2 Shandong Provincial Key Laboratory of High Strength Lightweight Metallic Materials, Advanced Materials Institute, Qilu University of Technology, Shandong Academy of Sciences, Jinan 250014, China

**Keywords:** Al-Zn-Mg-Cu alloy, Al_3_ (Sc, Zr) particle, mechanical properties, microstructure

## Abstract

Transition element microalloying is important for improving the properties of Al-Zn-Mg-Cu alloys. Nevertheless, along with its high costs, increasing Sc content generates a harmful phase, limiting the strength of the alloy. In this experiment, we reduced the amount of Sc added to a Zr-containing Al-Zn-Mg-Cu alloy by one order of magnitude. The microstructure and mechanical properties of the alloys were studied by means of tensile tests, field emission scanning electron microscopy (FESEM), and transmission electron microscopy (TEM). The findings indicate that the alloys’ mechanical properties were progressively enhanced with the increase in Sc content from 0 to 0.04%. After adding 0.04% Sc, the tensile strength and yield strength of the Al-Zn-Mg-Cu-Zr-Sc alloy increased by 20.9% and 24.3%, reaching 716 MPa and 640 MPa, respectively, and the elongation decreased, but still reached 12.93%. The strengthening mechanisms of the trace addition of Sc are fine grain strengthening and precipitate and disperse strengthening, and Al_3_(Sc, Zr) particles hinder the dislocation and grain boundary movement. Drawing on insights from other studies on Sc microalloying in Al-Zn-Mg-Cu alloys, this experiment successfully reduced the amount of Sc added by an order of magnitude, the alloys properties were improved, and the effect of strengthening remained good.

## 1. Introduction

Al-Zn-Mg-Cu (7xxx) alloys have become indispensable structural materials in the aerospace and automotive industries due to their low density, high strength and excellent machining properties [1,2,3,4,5]. Therefore, with the persistent emphasis on the improvement of material properties in these industries, more rigorous criteria have been established for the comprehensive properties of the Al-Zn-Mg-Cu alloy [1,5,6,7]. The Al-Zn-Mg-Cu alloy was first discovered by Wiebel in 1932, and since then, by adjusting the main alloy element content and adding other elements, a variety of new alloys have been developed, continuously improving on the original alloy’s variety and performance. In recent years, many researchers and research institutions have been dedicated to developing 7xxx-series aluminum alloys, including alloy composition adjustment, microalloying [3,4,6], and optimizing the heat treatment process [2,8,9], aiming to improve their performance by controlling the microstructure of the alloys.

In terms of alloy composition adjustment, the microalloying of trace transition elements or rare-earth elements into Al-Zn-Mg-Cu alloys can affect the precipitation of the alloy during the subsequent heat treatment process [9], which has a strong impact on their strength, plasticity, and toughness. Among them, zirconium (Zr) and scandium (Sc) are used as transition elements, and their applications in Al-Zn-Mg-Cu alloys have garnered widespread attention. The effect of Zr on 7xxx-series aluminum alloys is mainly reflected by grain refinement, aging strengthening, corrosion resistance improvement, and high-temperature performance enhancement. As an effective grain refiner, zirconium can refine the grain of aluminum alloys, mainly because the addition of Zr can increase the recrystallization temperature of the alloys, inhibiting the occurrence of the recrystallization process, helping to maintain a fine grain size, and improving the strength and plasticity of the alloys. At the same time, zirconium can promote the aging precipitation process, form a fine distributed precipitated phase, and improve the strength of the alloys. In addition, zirconium can improve the corrosion resistance of aluminum alloys and reduce their quenching sensitivity; hence, these alloys can maintain good mechanical properties in high-temperature environments. On the other hand, adding Sc to Al-Zn-Mg-Cu alloys and conducting microalloying and aging treatments can also increase the recrystallization temperature and inhibit the recrystallization process. At the same time, Sc can form a precipitated phase in the alloys. As an effective nucleating agent, Sc can promote nucleation by reducing the nucleation barrier, thus achieving the effect of grain refinement. Al_3_Sc particles with an L1_2_ ordered structure are formed, and these particles have good compatibility with the α-Al matrix [1]. This compatibility helps the alloys to significantly increase their strength while maintaining high ductility [3,9,10]. In addition, the presence of Al_3_Sc particles can effectively prevent the recrystallization of the alloys, hinder the dislocation and grain boundary movement, refine the grain, and further improve the alloy’s mechanical properties [7,11].

However, the limited availability and the expensive cost of scandium (Sc) elements significantly constrain the application of Sc-containing alloys in various industries [1,9,11,12]. The economic barriers associated with Sc make it a less feasible option for large-scale production, limiting its potential benefits across a broad spectrum of applications. In addition, studies have indicated that while the incorporation of Sc into alloys can effectively refine the grain size, and improve mechanical properties, this addition may induce a phase transformation. The θ phase (AlCu phase) may be transformed into a W phase (AlCuSc phase), which has an adverse effect on performance [13]. In addition to this, studies by V. V. Zakharov et al. [14] and XU et al. [15] have discovered that increased Sc in an alloy can lead to the formation of the harmful W phase through its combination with Cu. This finding underscores the complexity of alloy design when incorporating Sc, as the interaction between Sc and other elements can result in the formation of phases that are less desirable for the desired application. These findings emphasize the need for the careful and precise control of Sc content in alloys to avoid the formation of such detrimental phases, ensuring the alloys maintain their optimal performance and structural integrity.

In order to overcome these problems, researchers have begun to explore the compound addition of Sc and Zr in Al-Zn-Mg-Cu alloys to reduce the required Sc content. The compound addition of Sc and Zr has good potential contributions to the properties of 7xxx-series aluminum alloys. The addition of these two trace elements, by forming fine and evenly distributed Al_3_(Sc,Zr) particles, not only refines the grains, but also significantly improves the strength of the alloys. This effect of grain refinement is also crucial for improving the plasticity and toughness of the material, as it helps to prevent brittle fracture. In addition, these microalloyed elements can promote the aging precipitation process to form a more stable and fine precipitated phase, thus further strengthening the alloys. In terms of corrosion resistance, the addition of Sc and Zr helps to improve the surface properties of alloys, reduce the formation of corrosion pits, and extend the service life of the material. Although the price of scandium is relatively high, the improvements it allows for in the performance of aluminum alloys is significant, which makes scandium and zirconium a research hotspot in the field of aluminum alloy microalloying [3,9,11]. However, the amount of Sc added is often still over 0.1% or even more, and research on the addition of high trace amounts of Sc is not well understood.

Therefore, in order to avoid the formation of the above-mentioned harmful phase (AlCuSc (W)) in the microstructure of Al-Zn-Mg-Cu alloys, and considering the high price of Sc, in this study, an Al-Zn-Mg-Cu alloy containing 0.08% Zr was compounded with trace amounts of 0.02% and 0.04% Sc, and the alloy still showed a good strengthening effect. Its properties were improved, and at the same time, the amount of additional Sc was successfully reduced. Also, the related strengthening phase and strengthening mechanism before and after the addition of Sc were studied. This research is expected to provide a reasonable reference for the microalloying of Al-Zn-Mg-Cu alloys, so as to promote its application, reduce production costs and improve economic benefits.

## 2. Materials and Methods

Al-Zn-Mg-Cu-Zr and Al-Zn-Mg-Cu-Sc-Zr alloys were prepared according to the alloy compositions in Table 1. The actual compositions of the three alloys were detected using an optical emission spectrometer (Spectra Max, PE8000; Molecular Devices, San Jose, CA, USA); each alloy component was tested three times to ensure accuracy. The average of the results from the three tests along with the standard deviations of the samples are all shown in the parentheses of Table 1. The alloys were named 7050 (without Sc), 7050-0.02Sc, and 7050-0.04Sc (containing 0.02%Sc and 0.04%Sc, respectively).

**Figure 1:**
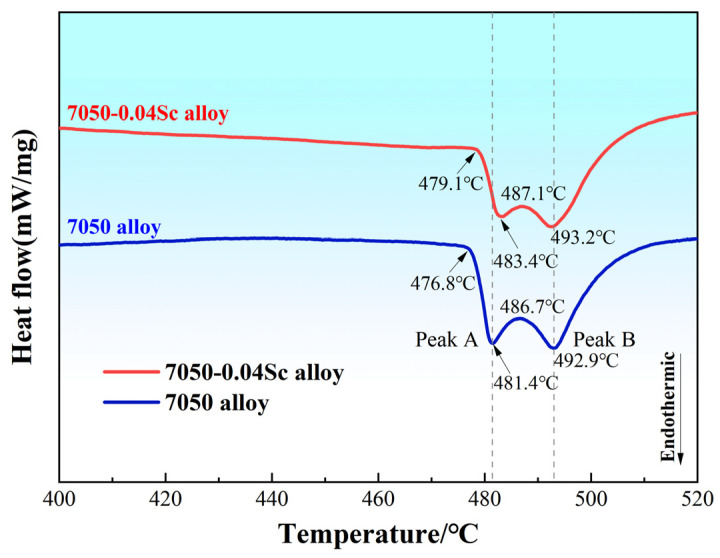
DSC curves of the studied alloys.

The experimental process was as follows: Pure aluminum (99.9%, all compositions are in wt%, unless otherwise stated), pure zinc (99.9%), pure magnesium (99.9%), pure copper (99.9%), Al-5Zr master alloy, and Al-2Sc master alloy were used as raw materials, and the alloys were melted in a resistance furnace with a graphite crucible. Then, the alloy melt was purified by 0.6% of C_2_Cl_6_ refining agent to remove slag and for degassing, and finally cast into a cylindrical graphite mold (Φ90 mm). The casting temperature of the alloy solution during casting was 720 °C and the mold was preheated to 300 °C. The initial solidification temperature and phase transition temperature of the alloys during the cooling process were analyzed using a differential scanning calorimeter (DSC, 404C, Netzsch, Selb, Germany). In total, 10–20 mg of the sample was weighed and placed in an Al_2_O_3_ ceramic crucible. Both the heating and cooling rates were set to 20 °C/min, and the test was conducted under an Ar atmosphere to prevent oxidation interference at high temperatures. Figure 1 shows the differential scanning calorimetry (DSC) curves of the as-cast alloy samples. As illustrated, there is a distinct endothermic peak A at 476–482 °C in the studied alloys with different Sc contents. The position of peak B is between 486 and 493 °C, which corresponds to the dissolution of the η phase and the S phase, respectively. For peak A, as the Sc content increases, the endothermic peak slightly shifts to the right, indicating that the studied alloys have a greater amount of non-equilibrium secondary phases, requiring more heat absorption during the melting process. Therefore, the homogenization temperature must be maintained below 475–482 °C to prevent overburning. After the ingots were held at 460 °C for 24 h for annealing treatment, hot extrusion treatment (HET) was performed at a temperature of 420 °C with an extrusion ratio of 30:1, followed by air cooling.

**Figure 2:**
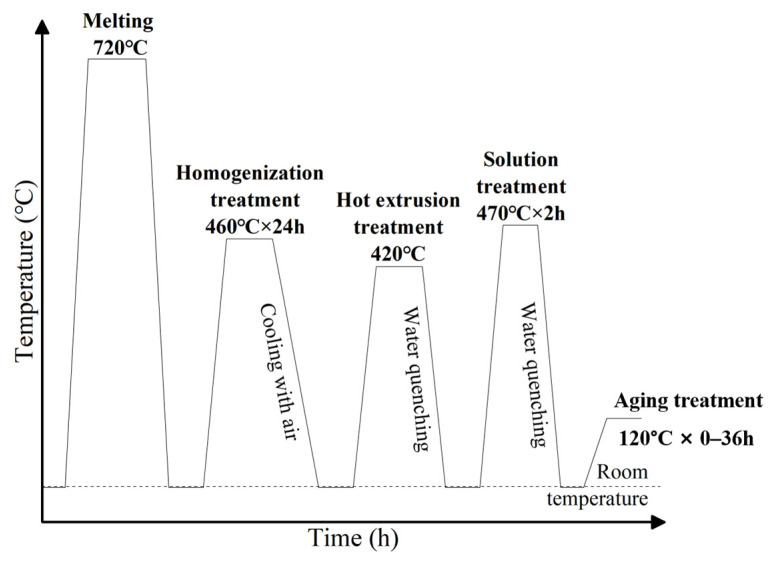
The heat treatment process flow diagram for this study.

Samples for room-temperature mechanical property analysis and microstructure observation were subjected to solution and peak aging treatment. The heat treatment process was as follows: solution treatment (ST) at 470 °C for 2 h, followed by quenching; aging treatment for the 7050 and 7050-0.02Sc alloys at 120 °C for 24 h, followed by air cooling; and for the 7050-0.04Sc alloy, aging treatment at 120 °C for 12 h, followed by air cooling [16,17,18,19,20,21,22]. The heat treatment process is schematically shown in Figure 2.

Microstructural observations and a compositional analysis of alloys with different Sc contents were conducted using a field emission scanning electron microscope (FESEM, SU-70; Hitachi, Tokyo, Japan) and an energy-dispersive X-ray (EDX, EX-250, Horiba, Kyoto, Japan) spectroscope. Samples were taken from the longitudinal section of the extruded rod at the center of the cross-section and were mounted, ground, and mechanically polished. After being etched with Keller reagent (1.0 mL HF + 1.5 mL HCl + 2.5 mL HNO_3_ + 95 mL H_2_O) for 15–20 s, the low-magnification microstructures of the alloys were observed using a metallography microscope (OM, Leica DM2700M, Wetzlar, Germany).

The grain size, grain orientation, and geometrically necessary dislocation density (GND) of the materials were analyzed using a field emission scanning electron microscope equipped with electron backscatter diffraction (EBSD, EDAX Velocity Super, Pleasanton, CA, USA), with an acceleration voltage of 20 kV. For EBSD analysis, the samples also required electrolytic polishing; the polishing solution was 10 vol% HClO_4_ + 90 vol% C_2_H_5_OH, with a solution temperature of 10–15 °C, a working voltage of 35 V, and a polishing time of 20 s. Bright-field (BF), dark-field (DF), high-angle annular dark-field (STEM-HAADF), and high-resolution (HRTEM) imaging and selected area electron diffraction (SAED) analyses were conducted using a high-resolution transmission electron microscope (HRTEM, FEI Talos F200x, Thermo Fisher Scientific, Waltham, MA, USA) equipped with an energy-dispersive X-ray (EDX, Oxford X-max 20, Zeiss, Oberkochen, Germany) spectroscopy probe to determine the morphology, composition, and structural information of the sample microregions. The acceleration voltage used was 200 kV.

**Figure 3:**
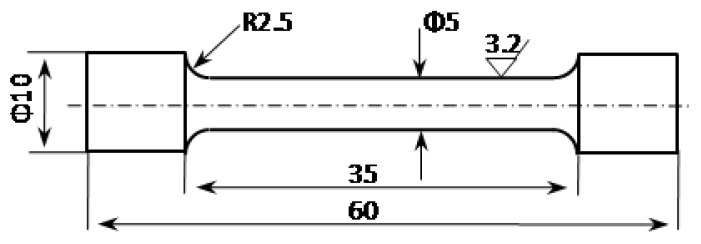
A tensile specimen schematic diagram for this study (unit:mm).

**Figure 4:**
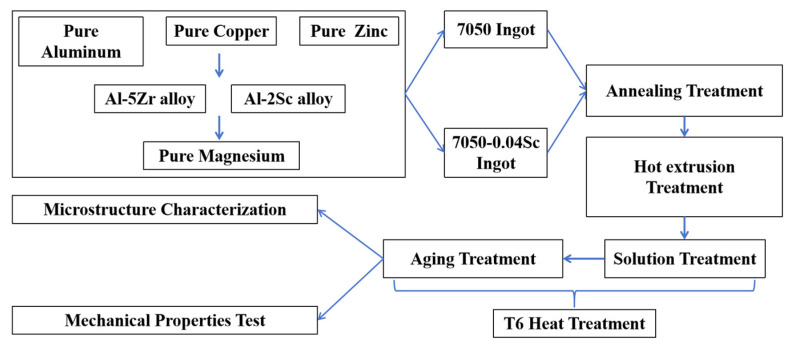
The process flow diagram for this study.

The samples were processed into tensile test rods according to the national standard GB/T 24196-2009, [23], machined according to Figure 3, with three rods prepared for each group. The room-temperature tensile properties of samples were tested on a universal testing machine (WDW-100D, Hai Rui, Jinan, China) at a strain rate of 2 mm·min^−1^, and the strength and elongation of the same sample were taken from the average values of the three rods. The hardness of the alloys was measured using a digital Brinell hardness tester (HBS-3000, Hua Yu Zhong Xin, Laizhou, China), and the thickness of the sample had to be less than 5 mm. The test force applied was 2452 N, and the holding time was 15 s. Each sample was tested in 3–5 positions, and the average values were taken as the results. The technical process of this study is shown in Figure 4.

## 3. Results and Discussion

### 3.1. Alloy Microstructure Characterization

**Figure 5:**
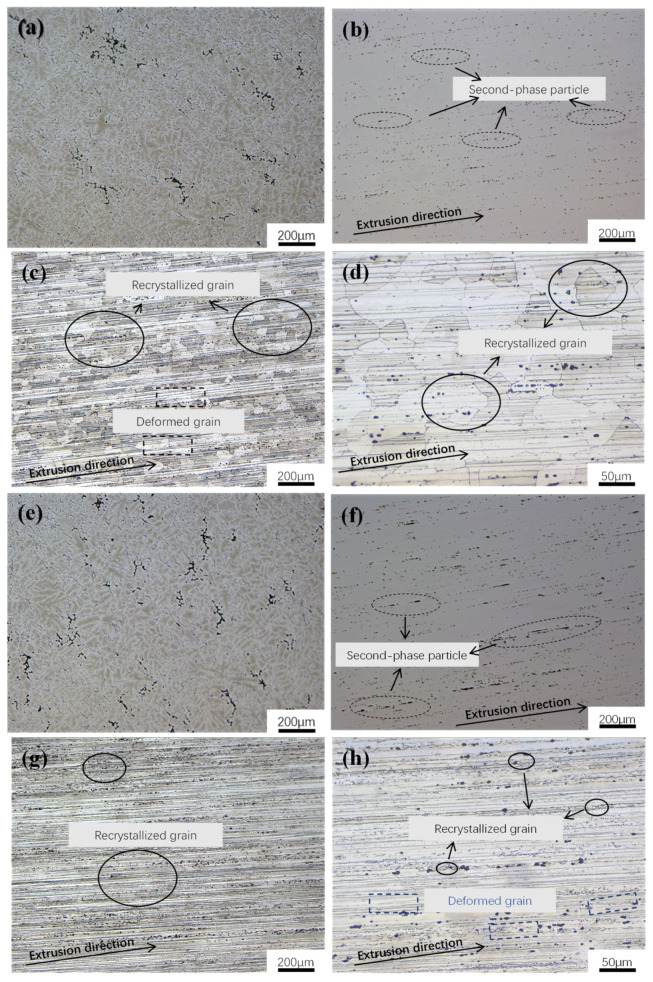
Metallographic photos of 7050 alloys with different Sc contents: (**a**–**d**) the 7050 alloy and (**e**–**h**) the 7050-0.04Sc alloy. In (**a**,**b**,**e**,**f**), the alloy is not etched, while in (**c**,**d**,**g**,**h**), the alloy is etched.

Figure 5 shows metallographic photos of the longitudinal sections of 7050 alloys with different Sc contents. Cast alloys exhibit a continuous or semi-continuous networked structure (Figure 5a,e), while the original grains of the 7050 and 7050-0.04Sc alloys are elongated in the extrusion direction after hot extrusion deformation (Figure 5b,f), exhibiting a typical fibrous structure. Meanwhile, the second-phase particles within the alloys are fractured and distributed in a chain-like pattern along the extrusion direction [10]. Further observation reveals that compared to the 7050 alloy, the 7050-0.04Sc alloy has more second-phase particles distributed along the extrusion direction, with a more uniform distribution and finer size, exhibiting a thinning trend from short, rod-like shapes to spherical shapes.

Upon observing the metallographic structure of the 7050 alloy after etching with Keller’s reagent (Figure 5c,d,g,h), the grain morphology of the alloy is clearer. In the metallographic structure of the 7050 alloy without Sc (Figure 5c,d), it can be seen that the grains of the alloy were coarse recrystallized grains with an average grain size ranging from 50 to 70 μm. The grain size was measured using the intercept method. However, after the trace addition of 0.04% Sc, there was a significant difference in the grain structure. The elongated strip-like deformed grains in the extrusion direction are more evident, as shown in Figure 5g,h. The large-sized recrystallized grains are almost no longer observable in the metallographic structure, and instead, many very fine recrystallized grains are present at the grain boundaries of the deformed grains along the extrusion direction. This indicates that the addition of Sc can effectively inhibit recrystallization in the alloy. The measurement shows that the average grain size of 7050-0.04 Sc ranges from 10 to 20 μm.

Studies have shown that Zener resistance caused by uniformly distributed precipitates is the main cause of grain boundary obstruction [10]. According to Zener theory [8], increasing the precipitates’ volume fraction-to-average radius ratio and maintaining a congruent interface between the precipitates and the matrix will increase the recrystallization resistance [24,25,26]. For second-phase particles with the same volume fraction, the recrystallization resistance will increase. Smaller particles can more effectively cause dislocations and hinder grain boundary migration, and this also corresponds to the fact that after peak aging (T6) heat treatment, as shown in Figure 5, the number of subcrystals in the 7050 alloy increases and the recrystallization rate increases, while in the 7050-0.04Sc alloy, recrystallization only occurs in a few areas and the deformed structure is still clearly retained.

**Figure 6:**
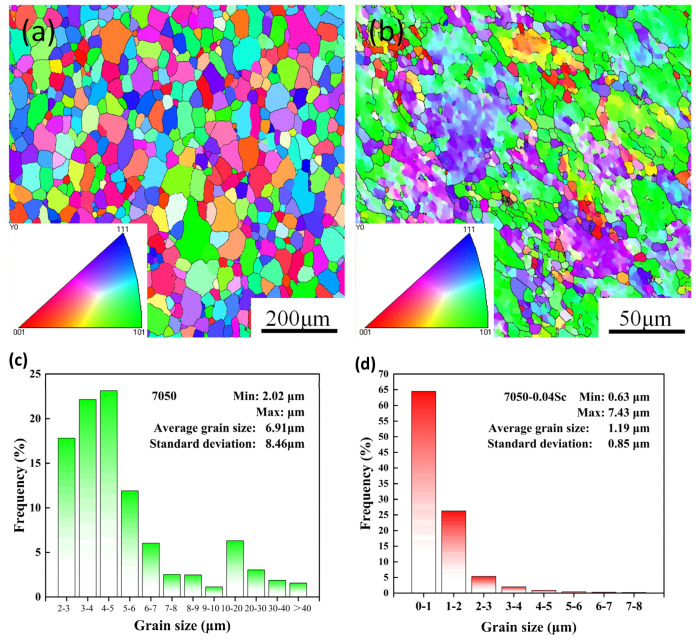
EBSD inverse pole figures of the surface of 7050 alloys with different Sc contents and grain size statistics: (**a**,**c**) 7050 alloy; (**b**,**d**) 7050-0.04Sc alloy.

To further investigate the effect of Sc on the recrystallization behavior of 7050 alloys, we used EBSD to analyze the microstructure of 7050 alloys with different Sc contents. Figure 6 shows the aluminum grain orientation distribution maps and the corresponding grain size distribution statistics of the 7050 alloy and the 7050-0.04Sc alloy. In the Euler triangle (inverse pole figure) shown in Figure 6a,b, red represents the <001>Al orientation, blue represents the <111>Al orientation, and green represents the <101>Al orientation. The grain orientation is determined by the color and distribution of the grains in the inverse pole figure.

In the subfigure of Figure 6a,b, the red, blue, and green colors represent the <001>, <111>, and <101> orientations of α-Al matrix, respectively, and we can find the grain orientations and distributions based on the colors in the inverse pole figures. From the color distribution, it can be concluded that on the cross-section of the as-extruded 7050 alloy, the α-Al grains are equiaxed, and there is no clear preferred orientation among the grains; <111>Al, <001>Al, and <101>Al orientations are all clearly observable. However, in the cross-section of the 7050-0.04Sc alloy, some deformed grain structures affected by extrusion can be observed, with a significantly preferred orientation toward the <101>Al direction. Additionally, it is observed that a small number of fine recrystallized grains formed on the boundaries of some larger deformed grains, which corresponds to the results observed in the metallographic photos in Figure 5e,f, and Figure 6c,d, which show the grain size statistics for the materials. The statistics reveal that the α-Al grain size in the 7050-0.04Sc alloy is smaller than that in the 7050 alloy. The average grain size of the 7050 alloy is 6.91 μm, while after the trace addition of 0.04% Sc to the alloy, the average grain size of the alloy decreases to 1.19 μm.

**Figure 7:**
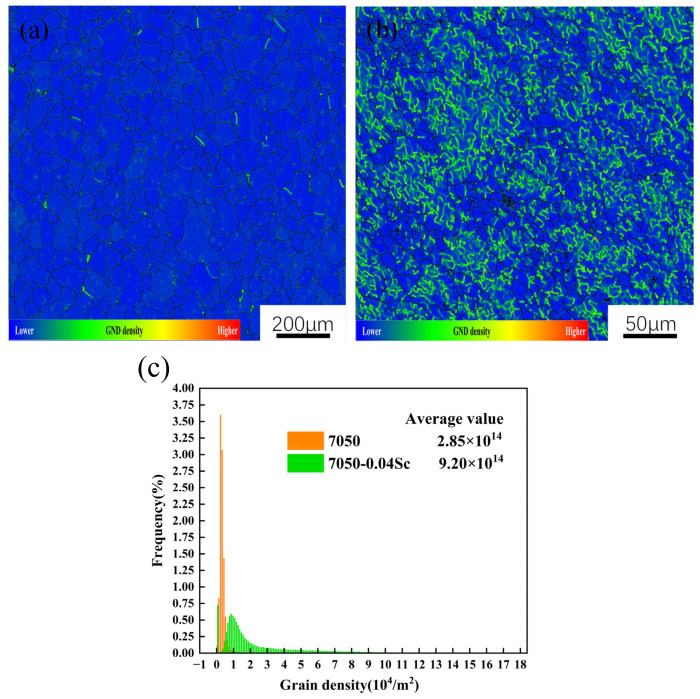
GND density distribution and its statistics for 7050 alloys with different Sc contents: (**a**) 7050 alloy; (**b**) 7050-0.04Sc alloy; (**c**) GND density statistics.

During the extrusion process, the material deforms to adapt to the applied external force applied. This deformation results in a rearrangement of the matrix crystal lattice to reduce energy and maintain a stable crystal structure. The spontaneous rearrangement of the matrix crystal lattice during extrusion leads to a significant increase in dislocations [2,10]. Figure 7 shows the geometrically necessary dislocation (GND) density distribution maps for alloys with different Sc contents, where the blue areas represent a low GND density while the red areas represent a high GND density. The average GND densities for the 7050 and 7050-0.04Sc alloys are 2.85 × 10^14^/m^2^ and 9.20 × 10^14^/m^2^, respectively, as shown in Figure 7c. This indicates that the 7050-0.04Sc alloy retains a higher dislocation density after T6 heat treatment, compared to the 7050 alloy; the 7050-0.04Sc alloy requires less driving force (lattice distortion energy) for recrystallization, and consequently undergoes less recrystallization.

Furthermore, as can be seen from Figure 7b, the grain boundaries in the high-density dislocation regions are of the small-angle type. The primary reason for this is the creation of Al_3_(Sc, Zr) particles within the alloy following the incorporation of minimal Sc. These particles can strongly pin the dislocations in the alloy, so the subgrain boundary cannot effectively absorb the dislocation. In addition, the low angle of the grain boundary morphology is maintained; thus, the recrystallization nucleation of the alloy is inhibited [27].

**Figure 8:**
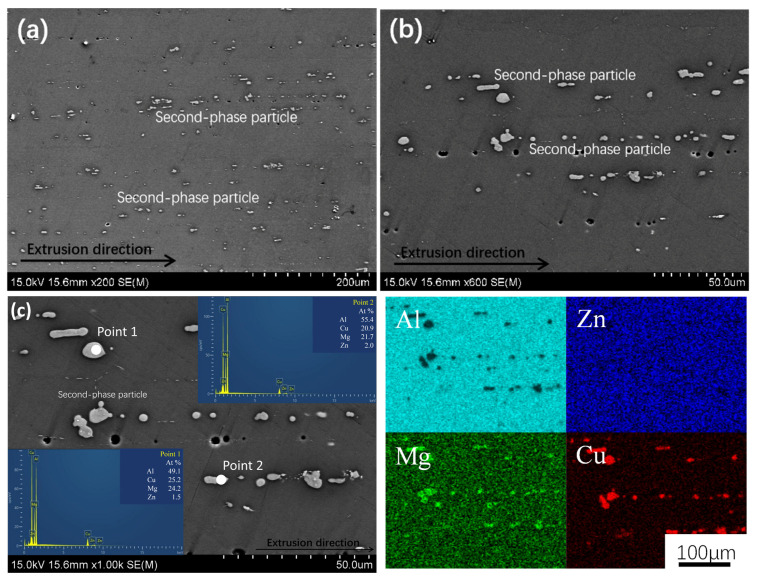
SEM photos with different magnifications (**a**) 200×, (**b**) 600×, (**c**) 1000×, and EDS analysis of 7050 alloy.

The SEM microstructure photos of the longitudinal section of the 7xxx alloy without added Sc, along with the EDS analysis results at various probe points and surfaces, are shown in Figure 8. It can be seen from Figure 8a,b that in the 7xxx alloy, the non-equilibrium phases that are distributed in short rod-like (approximately 10–20 μm in size) or spherical (approximately 1–2 μm in size) shapes along the extrusion direction have a coarse size and high density, and they are arranged in a streamline pattern. The EDS analysis result indicates that the Al:Cu:Mg ratio is approximately 2:1:1 (as shown in probe points 1, 2 in Figure 8c), which corresponds to the Al_2_CuMg (S) phase in the Al-Zn-Mg-Cu alloy [28]; by observing the surface distribution map of Zn, it can be seen that Zn is uniformly distributed within the matrix of the Al-Zn-Mg-Cu alloys. In addition, some black spherical shapes can be seen in the figure, and they appear as obvious defects inside the material, with smooth edges and a uniform spherical contour. Due to the relatively high concentration of Zn, Mg, and Cu in the alloys, these porosities were created in the matrix, which is typical of Al-Zn-Mg-Cu alloys [17].

**Figure 9:**
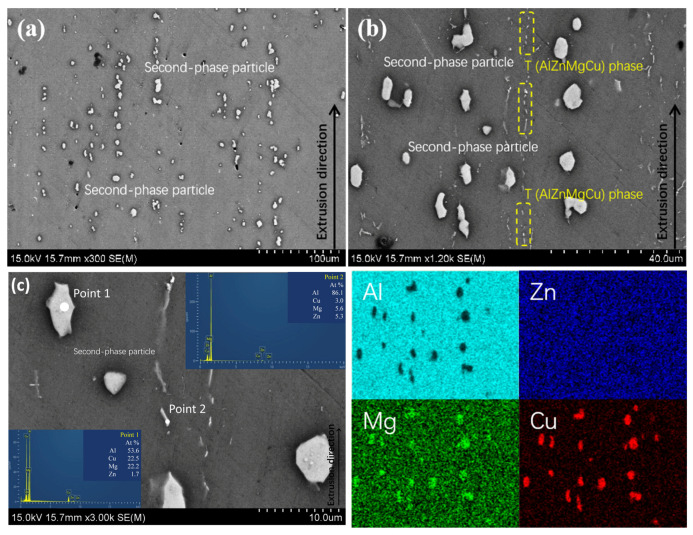
SEM photos with different magnifications (**a**) 300×, (**b**) 1200×, (**c**) 3000×, and EDS analysis of 7050-0.04Sc alloy (T (AlZnMgCu) phase is in the yellow box).

The SEM microstructure photos of the longitudinal section of the 7050-0.04Sc alloy with 0.04% added Sc, along with the EDS analysis results at various probe points and surfaces, are shown in Figure 9. By comparing Figure 8a,b and Figure 9a,b, it can be clearly observed that after adding the Sc element, the number of second-phase particles is higher, while the size decreases, the number of short, rod-like particles decreases, the number of spherical shapes increases, and the grains tend to become thinner. Through the surface analysis of Figure 9b and further point analysis of the phase in the locally amplified Figure 9c, it can be seen that the alloy mainly consists of coarse S phases (detection point 1) and the four-component eutectic T phase (AlZnMgCu) [29] (detection point 2). In Figure 9b, a phase with a significantly finer grain size, continuously distributed along the extrusion direction, is observed, as indicated in the yellow box, this presumably being a MgZn phase in the alloy. Overall, the results determined via SEM are consistent with the metallographic photos shown earlier, confirming that Sc has a refining effect on the grains in the microstructure. After the addition of Sc, the phases in the alloy microstructure tend to be more evenly distributed along the extrusion direction, their density increases, and the grains become finer.

As a heat-treatable, strengthening aluminum alloy, precipitation hardening is the most significant strengthening mechanism in 7xxx-series aluminum alloys after aging treatment. The Al-Zn-Mg-Cu-Zr alloy has a relatively fixed precipitation sequence during aging, which can be simply represented as SSS (supersaturated solid solution) → GP zones (coherent, rich in Zn) → metastable η’ phase (semi-coherent, MgZn_1-2_) → equilibrium η phase (incoherent, MgZn_2_) [30,31,32], with the η’ phase being the main strengthening phase in the alloy [33].

**Figure 10:**
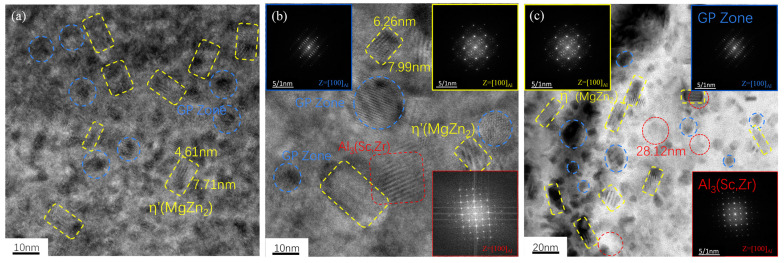
TEM images of 7050 alloys with different Sc contents after T6 heat treatment: (**a**) 7050 alloy; (**b**,**c**) 7050-0.04Sc alloy (The yellow rectangles represent MgZn_2_ phases, blue circles represent GP zones, red rectangles represent Al_3_(Sc,Zr) phases).

Figure 10a–c show microscopic organization photos of samples of the 7050 and 7050-0.04Sc alloys under a transmission electron microscope after T6 treatment. It can be seen that after T6 treatment, the alloys contain plate-like (indicated by blue circles), short, rod-like (indicated by yellow rectangles), and ball-like (indicated by red rectangles) precipitates. These precipitates are GP zones and η’ and Al_3_(Sc,Zr) phases. As the main strengthening phases of the Al-Zn-Mg-Cu-Zr alloy, the dimensions and arrangement of the GP zones and η’ phases have a direct influence on the mechanical properties of the alloy [34]. Measurements show the size of Al_3_(Sc,Zr) and η’ phases, and it can be seen that the Al_3_(Sc,Zr) phases are in the range of 20–30 nm, while the size of the η’ phase precipitated in the 7050 alloy before and after the addition of trace Sc is not significantly different, with a length of about 7–8 nm and a width of about 4–6 nm. Fourier transforms was performed to obtain diffraction spots, shown in the inserts in the upper left corner of Figure 10b for the GP zone and the lower right corner for the η’ phase, which demonstrates that the GP zone and η’ phase precipitate in the crystal of the 7050 aluminum alloy after peak aging (T6) heat treatment. In Al-Zn-Mg-Cu alloys, the primary strengthening phase is MgZn_2_ [35]. During the aging process, the supersaturated solid solution precipitates nanoscale MPt (GP zones, η’, and η), which contributes to the precipitation hardening of the alloy [34]. The precipitates impede the movement of dislocations and help to increase strength because dislocations must either shear through the precipitates or bypass them (Orowan strengthening) [36]. In both cases, additional stress is required to move dislocations over the slip planes, thereby increasing the material’s strength.

**Figure 11:**
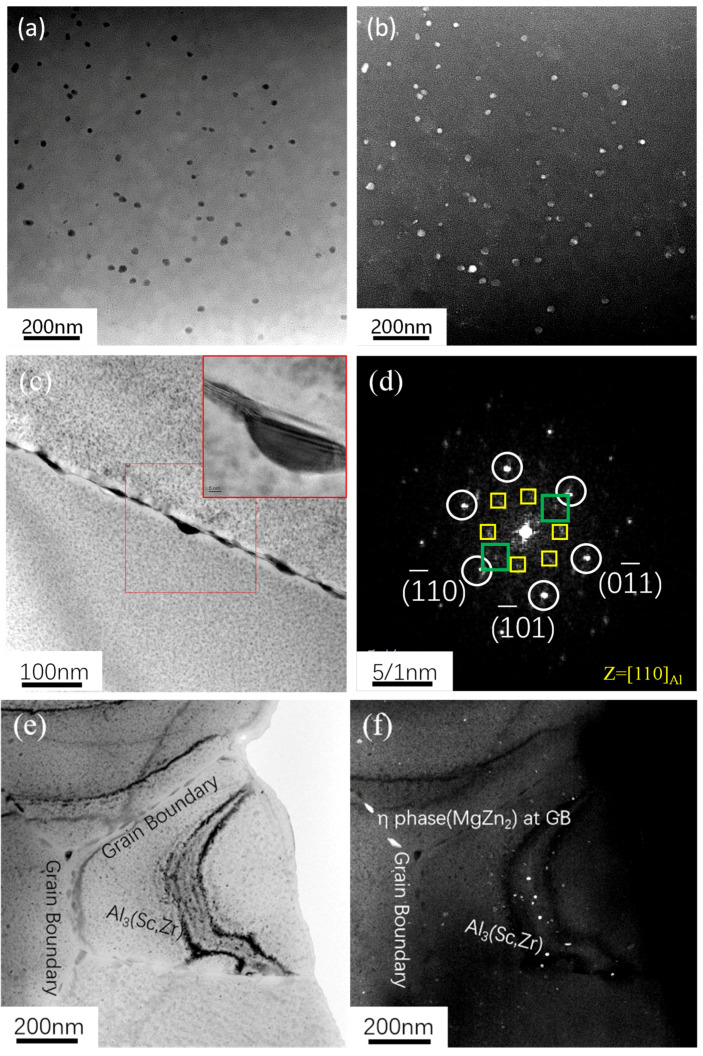

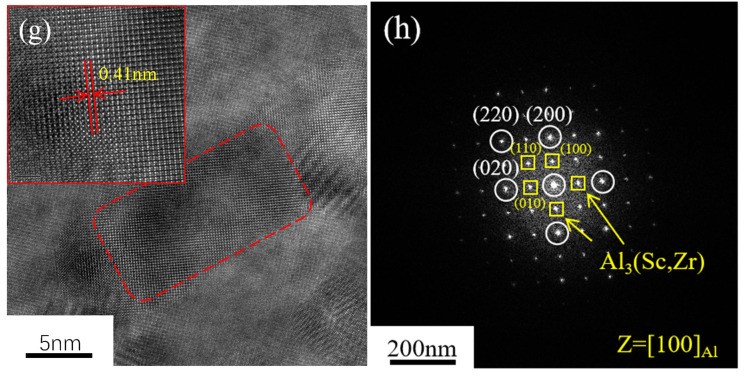
BF, DF, and HRTEM images of 7050 alloys with different Sc content after T6 heat treatment: (**a**–**d**) 7050 alloy; (**e**–**h**) 7050-0.04Sc alloy (Yellow boxes indicate Al_2_Zr particles while green boxes indicate Al_3_Zr particles in (**d**), yellow boxes indicate Al_3_(Sc,Zr) particles in (**h**)).

The dark-field images of the Al_3_Zr and Al_3_(Sc, Zr) precipitates in the 7050 and 7050-0.04Sc alloys are shown in Figure 11b,f. It can be observed that the Al_3_Zr and Al_3_(Sc, Zr) phases are uniformly dispersed in the aluminum matrix. In the bright-field image in Figure 11a, the particles are confirmed to be Al_3_Zr through Ashby–Brown contrast and the reflection of the upper structure. In the 7050 alloy, the grain size of the Al_3_Zr particles ranges between 40 and 50 nm, while the Al_3_(Sc, Zr) precipitates formed after adding trace 0.04% Sc have a smaller grain size, approximately 20–30 nm.

A large number of studies report that the addition of a small amount of Zr element to Al-Zn-Mg-Cu alloys results in the formation of spherical Al_3_Zr particles in the crystal during solid-solution and aging treatment [6,9,11,12,26,27,36,37,38,39,40,41]. These particles are coherent with the matrix, with a lattice constant of a = 0.405 nm. Figure 11c is a high-resolution image of a typical precipitate particle. Fourier transforms of the image resulted in the diffraction pattern shown in Figure 11d. However, the analysis of the diffraction pattern revealed that this particle is not the metastable Al_3_Zr particle described in the literature. After calibration of the diffraction pattern, the diffraction points of the matrix (indicated by white circles in the figure) and the diffraction spots of the Al_2_Zr particles (outlined by yellow boxes) were identified. In addition, very small and weak diffraction spots of Al_3_Zr particles are also present, marked by green squares. In contrast, after the addition of trace 0.04% Sc, the diffraction pattern in Figure 11h shows that the Al_3_(Sc, Zr) precipitates formed uniformly in the alloy matrix, maintaining a coherent relationship with the matrix and exhibiting an L1_2_-type structure. The interplanar spacing is approximately 0.41 nm, as shown in Figure 11. According to the literature, the interplanar spacing of Al_3_Zr and Al_3_Sc is the same, approximately 0.4101 nm [37]. This further confirms that these particles are indeed an Al_3_(Sc, Zr) phase.

The strengthening mechanisms of Al-Zn-Mg-Cu alloys include grain boundary strengthening, solid solution strengthening, dislocation strengthening, and precipitation dispersion strengthening. After adding Sc, the alloy grains are refined, resulting in grain boundaries with a high volume density that hinder the movement of dislocations into adjacent grains, strengthening the material. When other elements are alloyed with the metal matrix as solute atoms, solid solution strengthening occurs. The solute atoms are of a different size to the matrix, causing changes in the strain field and interacting with dislocations to increase the material’s strength. Regarding precipitation/dispersion strengthening, the spherical Al_3_(Sc, Zr) particles could pin the migration of dislocations during the hot extrusion process. They are distributed near the dislocation lines and subgrain boundaries. Compared to the 7050 alloy, the recrystallization process of the alloy is significantly inhibited after the addition of trace 0.04% Sc. This is mainly due to the smaller and coherent Al_3_(Sc, Zr) precipitates strongly hindering the growth of dislocations and grain boundaries. They pin dislocations and hinder the movement of dislocations to rearrange into subgrain boundaries and develop into small-angle grain boundaries, which further inhibits recrystallization nucleation. According to the dispersion strengthening theory [42], the increase in strength caused by the Al_3_(Sc, Zr) precipitates is controlled by the smaller size shear mechanism and the larger size Orowan dislocation bypass mechanism [32]. The critical precipitate radius in aluminum alloys is 2.1 nm; therefore, the Al_3_(Sc, Zr) precipitates with diameters from 20 nm to 30 nm strengthen the 7050 aluminum alloy through the Orowan bypass mechanism after aging treatment. According to the Orowan mechanism theory, the Orowan strengthening effect of the precipitates is approximately inversely proportional to the average precipitate spacing [12]. In this work, the average spacing of the secondary Al_3_(Sc, Zr) particles in the 7050-0.04Sc alloy is significantly smaller than that of the Al_3_Zr particles in the 7050 alloy; thus, the formation of the Al_3_(Sc, Zr) precipitates helps to refine the alloy grains and enhance the mechanical properties of the alloy, which further improves its thermal stability, consistent with the results of its mechanical properties.

**Figure 12:**
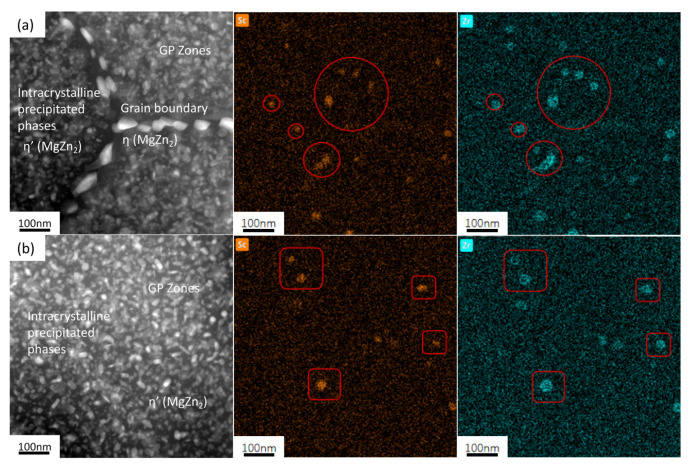
HAADF images of (**a**) grain boundary and adjacent grain interior (**b**) intracrystalline, and EDS element surface distribution of 7050-0.04Sc alloy.

Figure 12 shows the HAADF image and its element distribution map of the 7050-0.04Sc alloy, where it is found that the Zr at the core of the Al_3_(Sc, Zr) precipitate is replaced by Sc, forming a core–shell structure with a rich Sc core and a rich Zr shell [2,19,27,36,43]. The formation and coarsening of the precipitates are closely related to the diffusion rate of the dispersed forming elements. Sc diffuses rapidly in Al, and a literature review shows that the diffusion rate of Sc in Al is more than four orders of magnitude higher than that of Zr in Al at 300 °C. Therefore, Al_3_Sc dispersoids (cubic L1_2_ structure) can nucleate quickly and uniformly, but the high diffusivity of Sc also means that these dispersoids can coarsen relatively quickly. Via the cooperative effect of Sc and Zr, Al_3_(Sc, Zr) dispersoids are formed, which exhibit rapid nucleation, a uniform distribution, and slow coarsening. This is because the Zr hinders the diffusion of Sc at the interface, thus reducing the coarsening rate of the Al_3_Sc precipitates, which is consistent with the observed element distribution results.

The Al_3_(Sc, Zr) particles have a lower lattice mismatch with the matrix compared to the Al_3_Sc particles, allowing the former to maintain a larger coherent diameter [37]. Additionally, since the diffusion rate of Sc atoms is higher than that of Zr atoms at the same temperature, the thermal stability of the Al_3_(Sc, Zr) particles core–shell structure in the alloy is enhanced, enabling them to remain stable during subsequent solid solution and aging processes. This also explains why the Al_3_(Sc, Zr) precipitates exhibiting higher thermal stability than the Al_3_Sc precipitates. We can also verify this phenomenon from the aging hardness curve of the alloy in the following sections.

In Al-Zn-Mg-Cu alloys, defects such as dislocations and vacancies within the grains are eliminated during aging [44]. Therefore, only the grain boundaries (GBs) in the microstructure provide preferential nucleation sites for precipitates [45]. Precipitates firstly at the grain boundaries and continue growing. Then, they absorb solute atoms from the surrounding particles to convert into stable phases. After that, the nucleation of precipitates near the grain boundaries is suppressed, leading to the formation of a precipitation-free zone (PFZ) near the grain boundaries.

**Figure 13:**
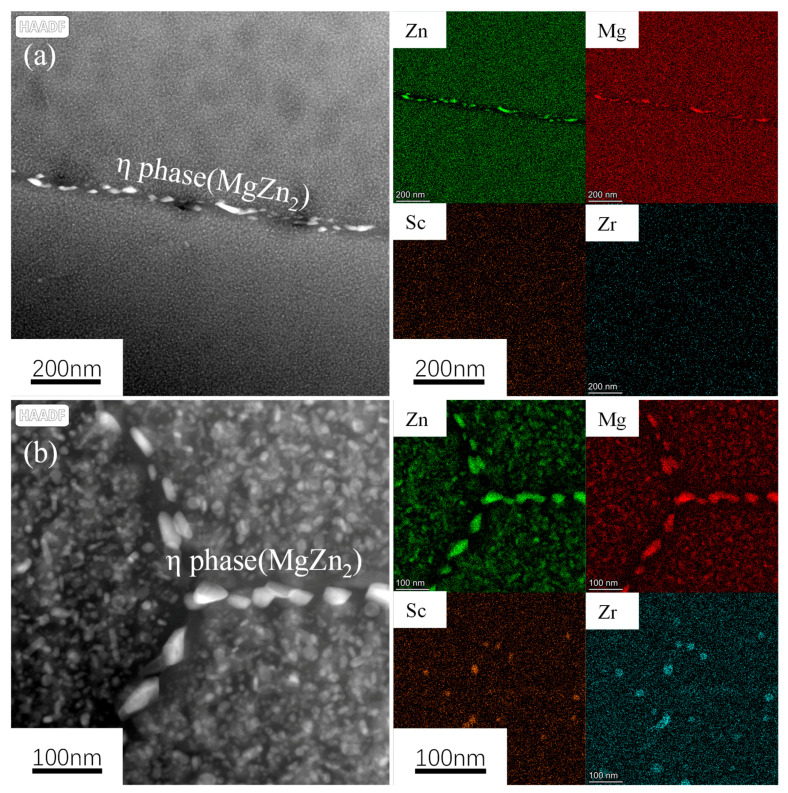

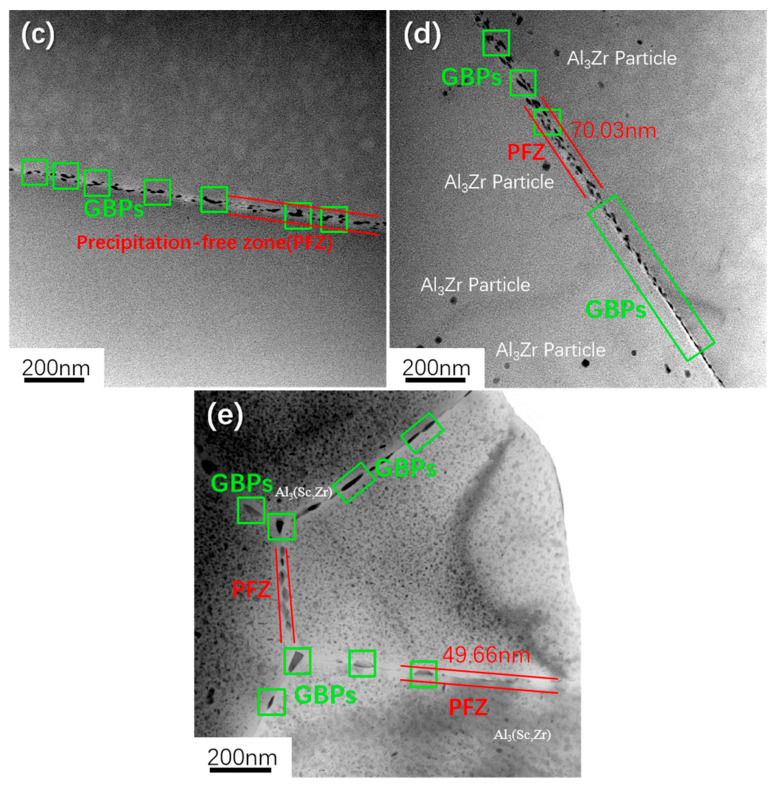
Grain boundary image and EDS element plane distribution map of 7050 alloys with different Sc contents: (**a**,**c**,**d**) 7050 alloy; (**b**,**e**) 7050-0.04Sc alloy.

Comparing Figure 13c–e, it can be seen that many η’ (MgZn_2_) precipitates are uniformly dispersed within the grains. However, at the grain boundaries of the 7050 alloy, as shown in Figure 13c,d, there are coarse rod-like continuous distributions of secondary precipitates (indicated by green rectangles). After the addition of trace 0.04% Sc, as shown in Figure 13e, the size of these precipitates at the grain boundaries decreases, and their distribution changes from continuous to discontinuous. As shown in the element distribution maps in Figure 13a,b, the precipitated secondary phase distributed at the grain boundaries is the equilibrium phase η (MgZn_2_). From Figure 13c–e, it can be seen that the width of the precipitation-free zone (PFZ) at the grain boundaries (indicated by red lines) in the 7050 alloy ranges between 60 and 70 nm. After the addition of Sc, the width of the PFZ also decreases significantly, reaching 49.7 nm. At the same time, it can also be observed that, whether Sc is added or not, there is almost no difference in the distribution and coarsening degree of the phases within the grains, indicating that the addition of Sc and Zr has almost no effect on the size, density, or distribution of the aging precipitates within the grains.

Comparing the distribution of Sc and Zr elements in Figure 13a,b, no discontinuous precipitation of Al_3_Sc dispersoids at the grain boundaries was observed. This indicates that when Zr is added alone, Al_3_Zr is mainly distributed within the grains of the alloy. However, the Al_3_(Sc, Zr) precipitates after the addition of Sc both within the grains and at the grain boundaries of the alloy, suggesting a more uniform distribution. Therefore, they can more effectively pin the grain boundaries and hinder their movement, thereby inhibiting recrystallization behavior.

The Sc-containing 7050 aluminum alloy used in this experiment retains the deformed streamlined structure and subgrain structures formed during deformation recovery after solid-solution and aging treatment [37]. For the grain structures that do not undergo recrystallization, grain boundaries with small angles will exist. Since the energy of small-angle grain boundaries is lower than that of large-angle grain boundaries and is close to the energy within the grains, the amount of the η phase precipitated at the grain boundaries is reduced and appears in a discontinuous distribution [26]. In addition, it is difficult to form precipitation-free zones (PFZs) along small-angle grain boundaries. The presence of PFZs in a microstructure usually reduces the grain boundary strength of the alloy [2]. However, due to the pinning effect of Al_3_(Sc, Zr) particles on the grain boundaries, the precipitation rates within and at the grain boundaries are not significantly different, resulting in relatively uniform aging precipitates. Therefore, a narrow PFZ is observed, which is conducive to better grain boundary bonding [2]. On the other hand, the pinning effect of Al_3_(Sc, Zr) precipitates on dislocations reduces the number of dislocations sliding towards grain boundaries (GBs), which lowers the stress concentration at the grain boundaries and avoids premature material fracture, thereby improving the plasticity of the alloy and achieving a good strength-to-plasticity match. At the same time, the existence of a PFZ at the grain boundary and the continuity of precipitate phases at the grain boundaries also directly affect the corrosion resistance of the alloy.

### 3.2. Alloy Mechanical Properties

**Figure 14:**
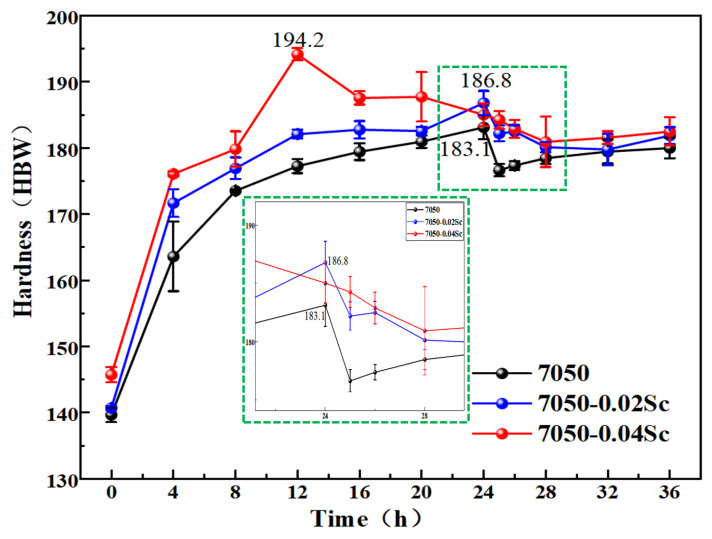
Aging hardness curves of alloys with different Sc contents.

Al-Zn-Mg-Cu-Zr alloys belong to the category of heat-treatable strengthening alloys. To determine the peak hardness of the alloys, different alloys with added Sc were subjected to aging treatment at 120 °C for 0–36 h after solid-solution quenching. Figure 14 shows the aging hardening curve of the measured alloy at 120 °C. From the figure, it can be seen that the hardness of the alloys increases rapidly in the early stage of aging, and the hardness growth starts to slow down after 4 h. The 7050-0.04Sc alloy reaches its hardness peak at 12 h, with a hardness value of 194.2HBW, while both the 7050 alloy and the 7050-0.02Sc alloy reach their hardness peaks at 24 h, with hardness values of 183.1HBW and 186.8HBW, respectively. As the aging time continues to extend, the hardness of the alloys begins to decrease, but the decrease is relatively small, indicating that the formation of Al_3_Zr and Al_3_(Sc,Zr) phases after adding Sc and Zr is beneficial to the improvement of the thermal stability of the alloys. According to research by D. C. Dunand [46,47], when the aging temperature of the alloy is increased from 350 °C to 400 °C, the rate of hardness decrease accelerates, further demonstrating the thermal stability of the L1_2_ precipitate phase. Compared to the alloy without the addition of Sc, after Sc is added, the hardness values of the alloy at each time point are higher than those of the 7050 alloy, and the hardness increases with the increase in Sc content.

The age-hardening mechanism of Al-Zn-Mg-Cu alloys is a process used to improve the mechanical properties of the material by controlling the dissolution of the second-phase particles in the alloy. Before aging, the alloy needs a solution treatment. The purpose is to dissolve the strengthening phase (such as MgZn_2_ or Al_2_CuMg) in the alloy into the matrix to form a supersaturated solid solution. Solution treatment is usually performed at high temperatures to promote the diffusion of solute atoms. After solution treatment, aging treatment, a slow process during which solute atoms are redistributed in the matrix, is carried out. During aging, a variety of precipitated phases are formed in the alloy, and the interaction between the precipitated phase and dislocation is the core mechanism of age hardening. By pinning the dislocation, the precipitated phase prevents the slip of the dislocation and increases the mechanical properties of the alloy. By comparing Figure 6 and Figure 7, it can be observed that after adding 0.04%Sc, the alloy has a higher dislocation density and a finer average grain size. These factors, through dislocation strengthening and grain refinement strengthening mechanisms, result in better mechanical properties, as shown in Figure 14.

**Figure 15:**
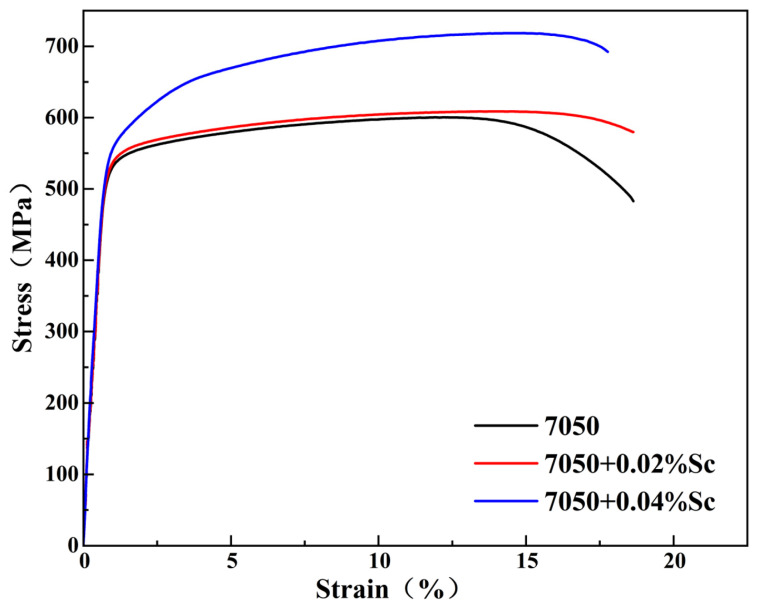
Stress–strain curves of 7050 alloys with different Sc contents in the peak aging state.

As a measure of a material’s resistance to the penetration of foreign objects into its surface, hardness cannot fully reflect the mechanical properties of the material. Therefore, we conducted tensile tests on the samples after peak aging treatment to obtain the stress–strain curves of the 7050 alloys with different Sc contents, as shown in Figure 15.

**Figure 16:**
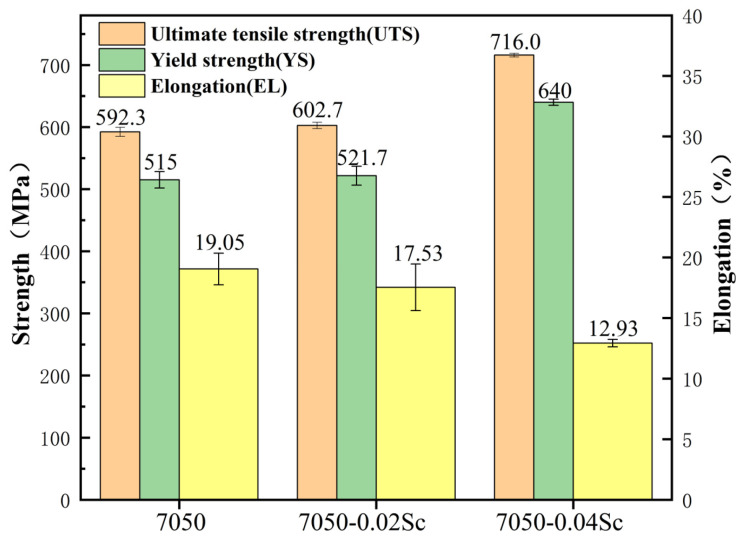
Mechanical properties of 7050 alloys with different Sc contents.

**Figure 17:**
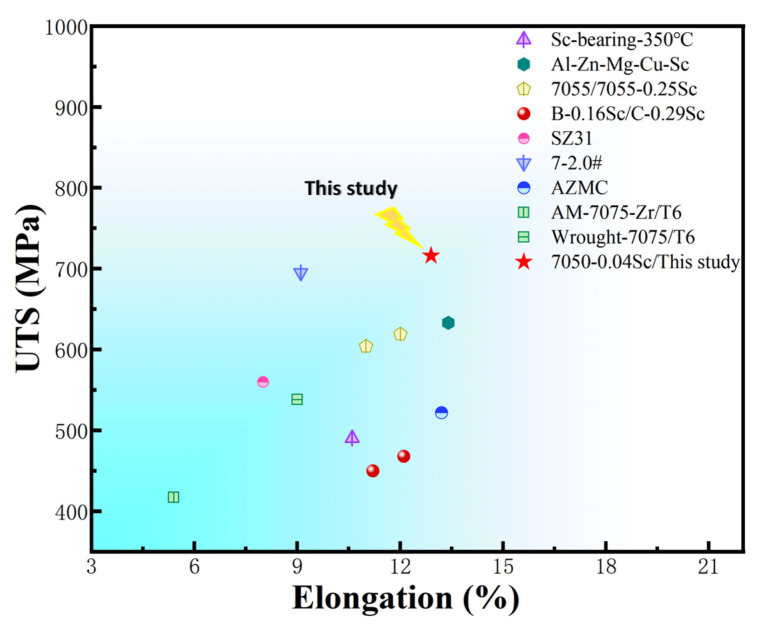
Tensile strength and elongation of the 7050-0.04Sc alloy in comparison with those of other reported 7xxx-series aluminum alloys.

The tensile properties of the alloys involved in the T6 heat-treated condition are shown in Figure 15 and Figure 16. In terms of tensile properties, with the increase in Sc content, the ultimate tensile strength of the alloy increased from 592 MPa (7050) to 716 MPa (7050-0.04Sc), an increase of 24.3%. The yield strength of the alloy increased from 515 MPa (7050) to 640 MPa (7050-0.04Sc), an increase of 20.9%. Apparently, the higher the Sc content, the higher the alloy strength, which is consistent with the hardness curves in Figure 14. The elongation gradually decreased with the increase in Sc content, but the elongation of 12.93% was still at a relatively high level. Therefore, it can be considered that the trace addition of 0.04% Sc can endow the 7050 aluminum alloy with good comprehensive properties. Figure 17 also shows that, compared with other reported alloys [1,2,7,12,16,17,24,48], the 7050-0.04Sc alloy can achieve both higher tensile strength and better elongation at the same time, achieving a good strength–plasticity match.

The reasons for the increase in strength mainly include two aspects [3]. (1) Firstly, they include fine grain strengthening, where the mechanical properties of the alloy are related to the grain size. The average grain size of the alloy decreases from 50 μm to 20 μm with the addition of 0.04% Sc, indicating a significant refinement effect of Sc on the 7050 alloy. The smaller averaged grain size, the greater the contribution to strength. The Al_3_(Sc, Zr) phases are distributed near dislocation lines and subgrain boundaries, inhibiting the nucleation and growth of recrystallization [19,24]. This relative refinement of the grains helps to increase the strength of the alloy. At the same time, the reduction in average grain size also helps to maintain the elongation. (2) Secondly, precipitation strengthening occurs, where the Al_3_(Sc, Zr) phases increase the grain boundary area of the alloy by pinning dislocations and refining the grains [26], thereby providing more preferred nucleation sites for precipitates. The expansion of nucleation sites promotes the nucleation of η’ phases. At the same time, it limits their growth and transformation into η phases, thereby increasing the strength of the alloy. This is also consistent with the phenomenon observed in the transmission electron microscope images in Figure 11e,f, where the Al_3_(Sc, Zr) precipitates pin dislocations; the results of grain boundary precipitation-free zones (PFZs) and grain boundary precipitates (GBPs) are shown in Figure 13c–e.

In summary, the microstructure of the alloys comprises strengthening precipitates (GP zones, η’, and η), Al_3_Zr and Al_3_(Sc, Zr) dispersoids, coarse intermetallic particles of Al_7_Cu_2_Fe, and a T phase (AlZnMgCu), as well as grain boundary precipitates (GBPs) and precipitate-free zones (PFZs). Due to this complex microstructure, a variety of microstructural mechanisms are at play during the performance processes of these high-strength aluminum alloys [49].

## 4. Conclusions

In this study, due to the limitations of using Sc, we innovatively reduced the amount of Sc by one order of magnitude to 0.02% and 0.04%, and found that this amount still has a good strengthening effect on the alloy.

(1) During the aging process, the hardness of the alloys with added Sc is greater than that of the 7050 alloy at various time points. The peak hardness of the 7050-0.04Sc alloy reaches 194.2HBW, and its tensile strength, yield strength, and elongation are 716 MPa, 640 MPa, and 12.93%, respectively, which indicate strong plastic matching.
(2) The addition of Sc effectively inhibits the recrystallization of the 7050 alloy. After Sc is added, the original coarse recrystallized grains disappear and become fine recrystallized grains distributed around deformed grains, while the average grain size decreases from 50 μm to 20 μm. At the same time, Sc refines the precipitated phase at the grain boundary (GB) of the alloy, reduces the width of the PFZ at the grain boundary, and improves aging’s strengthening effect.
(3) In the 7050 alloy, in addition to normal Al_3_Zr, there is an AlZr phase that is not coherent with the matrix, but after adding Sc, Al_3_(Sc, Zr) can form stably, and the strengthening effect is better. On the other hand, the distribution of the GP region and η phase in the grains is not affected by Sc.
(4) The strengthening mechanisms include grain boundary strengthening, solid-solution strengthening, and dislocation strengthening. The main precipitated phases of the studied alloy after T6 heat treatment are the η’ phase, GP zone, and Al_3_Zr or Al_3_(Sc, Zr) phase, while the main phase on the grain boundary is the η phase. As for the Al_3_(Sc, Zr) particles, there is an η’ phase and a GP zone, and the strengthening effect is exerted through the Orowan bypass mechanism.

## Figures and Tables

**Table 1:** Nominal compositions of Al-Zn-Mg-Cu-Sc-Zr alloys.

Alloy No.	Mass Fraction/%					
	Zn	Mg	Cu	Zr	Sc	Al
7050	6.2 (5.90, 0.022)	2.3 (2.37, 0.021)	2.3 (1.92, 0.047)	0.08 (0.072, 0.001)	0	Bal.
7050-0.02Sc	6.2 (5.89, 0.022)	2.3 (2.33, 0.022)	2.3 (1.90, 0.017)	0.08 (0.072, 0.002)	0.02 (0.018, 0.001)	Bal.
7050-0.04Sc	6.2 (5.57, 0.021)	2.3 (2.19, 0.024)	2.3 (1.79, 0.029)	0.08 (0.084, 0.001)	0.04 (0.033, 0.001)	Bal.

## Data Availability

The original contributions presented in this study are included in the article. Further inquiries can be directed to the corresponding author.
